# A Cu(II)-Based Fluorescent Probe for Carbon Monoxide, Nap-BC-Cu(II), Does Not Selectively Detect Carbon Monoxide

**DOI:** 10.3390/molecules31030415

**Published:** 2026-01-26

**Authors:** Dongning Liu, Hongliang Li, Shivanagababu Challa, Binghe Wang

**Affiliations:** Department of Chemistry and Center for Diagnostics and Therapeutics, Georgia State University, Atlanta, GA 30303, USA

**Keywords:** fluorescent probes, carbon monoxide, chemoprobes, chemosensing, optical probes

## Abstract

Reports of carbon monoxide (CO) pharmacology have spurred intense interest in developing its fluorescent probes with much success. However, one unfortunate event in this area is the wide-spread use of chemically reactive metal/BH_3_-CO complexes as “CO-releasing molecules” or CORMs that do not produce CO or produce CO in an idiosyncratic fashion. Consequently, a large number of reported fluorescent “CO probes” only respond to the CORM used, but not to CO. Though most of these issues have been clarified in the literature, there is a surprising recent publication on a Cu(II)-based fluorescent “CO probe,” Nap-BC-Cu(II), relying on undefined chemical principles. We reassessed the ability for Nap-BC-Cu(II) to detect CO and found no evidence for Nap-BC-Cu(II) to selectively detect CO at even non-physiologically relevant high concentrations (high micromolar) of CO. Marginal effects were observed only when CO was continuously bubbled through the “probe” solution for 15 min. Further, Nap-BC-Cu(II) was found to be sensitive to ascorbic acid and cysteine. Overall, this probe did not respond to CO in a pathophysiologically relevant context. Our findings do not support the notion of Nap-BC-Cu(II) being a CO probe for studying CO biology. We hope this will be the last of this saga of “CO probes” that do not afford selective detection of CO, largely due to the confusions caused by using chemically reactive CORMs.

## 1. Introduction

Contrary to its public perception of being a toxic agent at any level, the human body produces carbon monoxide (CO) at a sufficiently high rate, leading to high micromolar concentrations of CO in the blood in the hemoglobin-bound form, COHb, under normal physiological conditions [1,2,3,4,5,6,7]. CO has been shown to have organ-protection and anti-metastatic effects, among others [8,9,10]. Related to the study of CO biology, there is a need for the development of sensitive, selective, and real-time detection methods applicable under physiological conditions [11]. Among the detection modalities explored, fluorescent probes have emerged as a particularly attractive method [11,12,13]. Probes based on palladium-mediated reactions have found wide-spread applications through carbonylation [11], de novo fluorophore construction [13], and Tsuji–Trost-type reaction [14]. Since the first CO fluorescent probe published in 2012 [11], more than 100 publications have appeared reporting CO fluorescent probes [12].

**Scheme 1:**
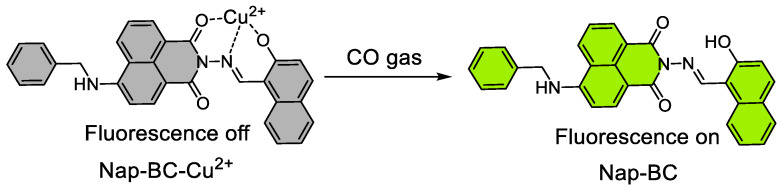
A schematic representation of the sensing mechanism for Nap-BC-Cu(II), as proposed by the original publication [21].

In developing fluorescent CO probes, commonly used CO sources include CO gas, metal–carbonyl “CO-releasing molecules” (CORMs) such as CORM-2 and CORM-3 [15], or metal-free CO prodrugs [16]. A valid probe should detect CO independent of its source—after all, CO is CO. Because several widely used CORMs do not reliably produce CO under typical experimental conditions and exhibit extensive CO-independent chemical reactivity [17,18,19,20], the use of such CORMs as the CO source has led to reports of fluorescent probes for CO that only detect the CORM used, but not CO itself [12]. Though most of these issues have been clarified in the literature [12], there is a surprising recent publication in 2024 by Fang and colleagues on using Cu(II)-based fluorescent “CO probe,” Nap-BC-Cu(II), relying on undefined chemical principles (Scheme 1) [21]. This publication recognizes the inability for an earlier Cu(II)-based system, DPHP-Cu(II), to detect CO [12,22,23] and specifically states the ability for a Nap-BC-Cu(II) mixture to afford “recognition specific for CO.” In this study, fluorescence quenching was attributed to a proposed 1:1 complexation between Nap-BC and Cu(II), while fluorescence recovery upon CO exposure was interpreted as resulting from the reduction in Cu(II) to Cu(I). CO reduction in Cu(II) typically requires elevated temperatures or a catalyst [24,25,26]. Therefore, the stated CO sensing ability and specificity were intriguing.

We sought to independently replicate and critically evaluate the sensing performance of the Nap-BC-Cu(II) probe. We synthesized the Nap-BC ligand and reconstituted the Nap-BC-Cu(II) mixture using reported conditions. Our results confirm the ability for Cu(II) to efficiently quench the fluorescence of Nap-BC. However, the Nap-BC-Cu(II) system is sensitive to reducing species such as vitamin C and thiol species (e.g., cysteine, glutathione) with about 70–100% restoration of the original fluorescence. In contrast, incubation with a large excess of CO or less than five equivalents of CORM-3 led to no meaningful fluorescence changes. Incubation with a large excess of CORM-3 (>10 equiv) led to a maximum of 20% fluorescence recovery. Under a non-physiologically relevant condition via bubbling pure CO continuous for 60 min, a significant fluorescent recovery was observed. Though the response to bubbling CO may suggest the likelihood of weak interactions of CO with Nap-BC-Cu(II), our findings do not support meaningful sensitivity or selectivity of the Nap-BC-Cu(II) system in sensing CO in the context of studying CO biology. We hope this will be last of this saga of “CO fluorescent probes” that do not selectively sense CO, because of the heavy reliance on chemically reactive CORMs as “CO surrogates” and the unprecedented chemistry sensing principle. Below we present the details.

## 2. Result and Discussion

### 2.1. Synthesis and Structural Confirmation of Nap-BC

As the first step of the validation work, we synthesized Nap-BC following the original literature procedures [21]. The product was characterized by using mass spectrometry and ^1^H- and ^13^C-NMR through comparison with the literature data (Figures S1 and S2). Our work confirms the structure of Nap-BC as stated in the original publication.

### 2.2. Confirmation of Literature Findings

**Figure 1:**
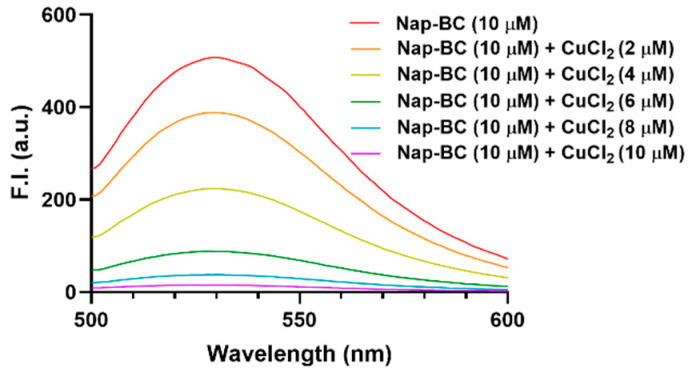
Fluorescence responses of Nap-BC (10 μM) in CH_3_CN/H_2_O (*v*/*v* = 4:1) solution to the addition of Cu(II) at varying concentrations. Briefly, the cuvette containing Nap-BC was vortexed and incubated at room temperature for 5 min after addition of Cu(II). Then, fluorescence reading was recorded (λ_ex_ = 445 nm, slit width = 5 nm).

As the first step in assessing this CO probe, we were interested in confirming the literature findings of the spectroscopic properties of the Nap-BC, its Cu(II) mixture and the subsequent response to CORM-3. We first studied the spectroscopic properties of Nap-BC (Figure 1 and Figure S3A). The UV-vis spectra of Nap-BC in CH_3_CN-H_2_O (*v*/*v*, 4:1) showed a λ_max_ of 445 nm. Similarly, the fluorescence spectra showed an emission peak at 535 nm. All such results are in agreement with that of the original publication, serving as secondary validation of the literature findings in this regard. 

Next, we examined the spectroscopic responses of Nap-BC to Cu(II). In the orignial publication (Scheme 1), Cu(II) was proposed to interact with Nap-BC, leading to fluorescence quenching. When increasing concentrations of Cu(II) were added to a fixed concentration of Nap-BC (10 μM) in CH_3_CN/H_2_O (*v*/*v* = 4:1), fluorescence intensity at 535 nm decreased progressively, indicating a strong quenching effect (Figure 1). Notably, the fluorescence was almost completely quenched upon the addition of 1.0 equivalent of Cu(II). Such results serve as secondary confirmation of the effect Cu(II) has on Nap-BC, as reported in the original publication.

**Figure 2:**
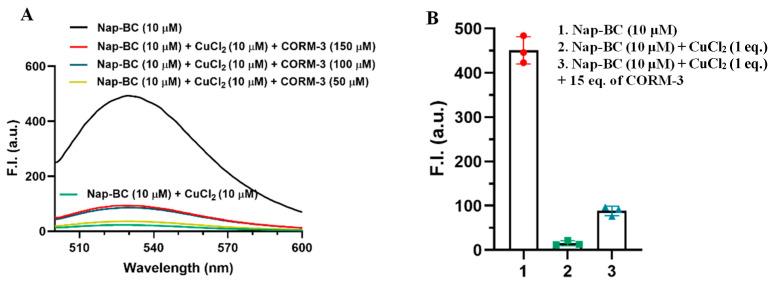
(**A**) Fluorescence responses of the Nap-BC-Cu(II) system (10 μM) to 50 μM, 100 μM, and 150 μM of CORM-3. (**B**) Comparison of fluorescence intensity of Nap-BC-Cu(II) + 15 eq. CORM-3, Nap-BC only, and Nap-BC-Cu(II) were used as controls. (*n* = 3, mean ± SD, λ_ex_ = 445 nm, λ_em_ = 535 nm, slit width = 5 nm, each dot represents an original data point).

Next, we examined the spectroscopic responses of the Nap-BC-Cu(II) solution to CORM-3. When 5, 10, or 15 equiv of CORM-3 was added to the Nap-BC-Cu(II) solution (10 μM, each), fluorescence restoration plateaueed at around 20% at 10 equiv (100 μM, Figure 2A,B). In contrast, the original publication described 75% fluroescence restoration. We are not sure about the reason for the quantitative difference between our results and that of the original publication. It should be noted 100 μM of free CO is beyond physiological relevance. Therefore, any findings beyond this concentration of free CO do not have meaningful implications in the context of understanding CO biology.

### 2.3. Response to CO Gas

**Figure 3:**
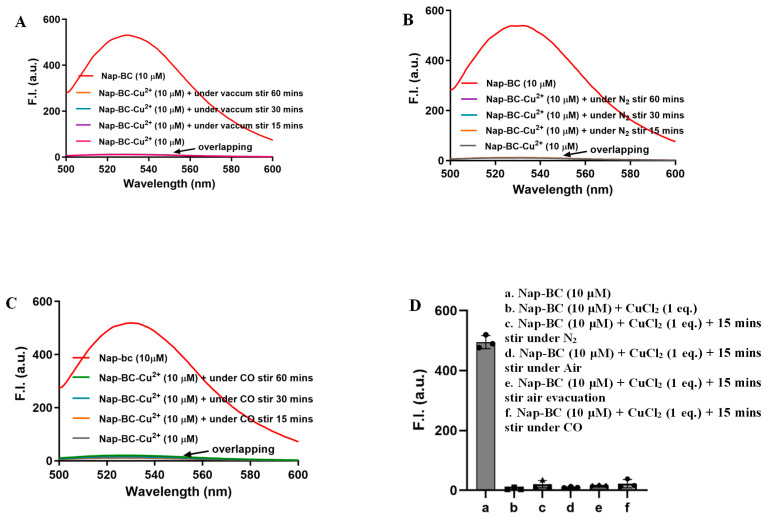
Fluorescence responses of the Nap-BC-Cu(II) system (10 μM) with stirring for up to 60 min under different conditions. (**A**) Vacuum, (**B**) N_2_, (**C**) CO. (**D**) Comparison of the fluoresce intensity of 15 min stirring under different atmospheres, Nap-BC only and Nap-BC-Cu(II) were used as controls. (*n* = 3, mean ± SD, λ_ex_ = 445 nm, λ_em_ = 535 nm, slit width = 5 nm, each dot represents an original data point).

To assess the key question of the ability for Nap-BC-Cu(II) to sense CO, we re-evaluated the probe’s response to CO in a balloon [27]. Specifically, 2 mL of Nap-BC-Cu(II) solutions were prepared in 20 mL vials, evacuated under vacuum, and then filled with a ballon of 250 mL of pure CO gas, which is ~10.2 mmol or about 1 million-fold excess in total amount and about 100-fold excess in solution concentration given the CO solubility of 1 mM. The solutions were stirred at 800 rpm for 15 min at room temperature, before fluorescence recording. N_2_ was used as a negative control. As shown in Figure 3A–D, no significant fluorescence differences were observed among the various atmospheric conditions (air, after air evacuation, N_2_, or CO) at the 15 min time point. It should be noted that CO quickly escapes in an open system, which is essentially all the cases in cell-culture experiments [28]. A lack of any response within 15 min foretells the inability to detect CO in the context of studying CO biology.

**Figure 4:**
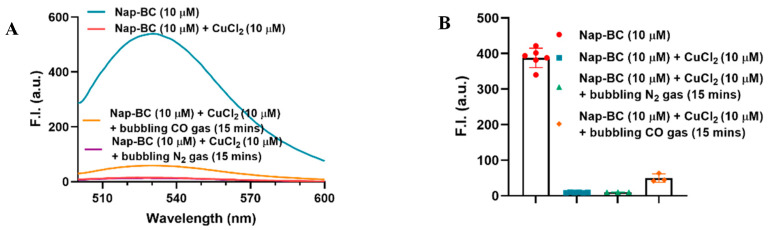
(**A**) Fluorescence responses of the Nap-BC-Cu(II) system (10 μM) under bubbling CO or N_2_ gas for 15 min. (**B**) Comparison of fluoresce intensity of 15 min bubbling CO or N_2_ gas, Nap-BC only, and Nap-BC-Cu(II) were used as controls. (*n* = 3, mean ± SD, λ_ex_ = 445 nm, λ_em_ = 535 nm, slit width = 5 nm, each dot represents an original data point).

Subsequently, we employed more forcing conditions by bubbling CO gas into the probe solution (10 μM) under ambient conditions for 15 min, providing an overwhelming excess of CO. As shown in Figure 4A, about 10% fluorscent restoration was observed. It is important to note the smaller magnitude of fluorescent restoration by CO as compared to CORM-3, even though the concentration of CO is much higher, indicating the CO-independent effect of CORM-3 [28]. As a comparision, N_2_ bubbling only led to a slight increase in the fluorescent signal. Though small, there is a clear difference between N_2_ and CO bubbling. When CO gas was bubbled into the Nap-BC-Cu(II) solution for 60 min (Figure S4), it showed around 80% fluorescence recovery of the orginal fluorescence intensity. Setting aside the question of whether this system is appropriate for CO detection, there is clearly some interaction between CO and the Nap-BC-Cu(II) mixture.

From these two types of CO gas experiments, we can conclude that simple CO exposure in large excess did not lead to meaningful fluorescence changes. However, direct bubbling of a large excess of CO gas or incubation with CO saturated solution for a long time (more than 15 min) did lead to some turn-on effects of the Nap-BC-Cu(II) system, suggesting some interesting chemistry (Figures S4 and S5). However, we should also note that even a small percentage of impurity in the CO gas may actually be a large amount after bubbling CO for 60 min. Therefore, there is a likelihood that it was not CO that led to the fluorescent changes observed. Because there is no practical meaning for the Nap-BC-Cu(II) system to respond to bubbling CO for 60 min in the context of studying CO biology, we did not pursue this matter further.

### 2.4. Findings About This Probe Using Reducing Agent and Thiol Species

**Figure 5:**
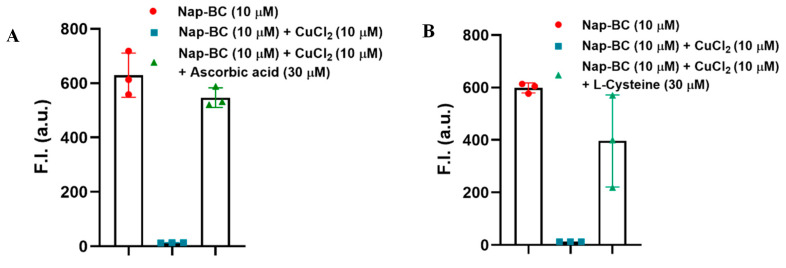
Fluorescence responses of the Nap-BC-Cu(II) system (10 μM) to (**A**) 30 μM of ascorbic acid, (**B**) 30 μM of L-cysteine. (*n* = 3, mean ± SD, λ_ex_ = 445 nm, λ_em_ = 535 nm, slit width = 5 nm, each dot represents an original data point).

Because the Nap-BC-Cu(II) system was described as affording “recognition specific for CO,” we assessed the effect of commonly seen molecules, which should be considered in assessing “specificity.” As examples, we examined the effects of a biologically relevant reducing agent, ascorbic acid (vitamin C), and L-cysteine under the same conditions. Specifically, the addition of 30 μM Vc to a Nap-BC-Cu(II) solution (10 μM) led to around 80% restoration of the Nap-BC-Cu(II) fluorescence (Figure 5A). The addition of L-cysteine (30 μM) also led to fluorescent intensity restoration of about 70% (Figure 5B), which is similar to the results reported in the original publication. Also, as shown in Figure S5B, 100 μM of glutathione (GSH) led to significant fluorescence restoration. It is important to note that such fluorescence responses are much higher in magnitude than that of CO, indicating intractable interference issues for CO in vivo detection, especially considering the fact that vitamin C exists in micromolar concentrations (around 28–85 μM) [29]; L-cysteine is present at high micromolar concentrations in the blood [30,31]; and GSH is present in the mM range [23].

## 3. Experimental Section

### 3.1. Material and Instruments

Chemical reagents were purchased from Sigma-Aldrich (Saint Louis, MO, USA) and/or Oakwood (Estill, SC, USA). Solvents were purchased from Fisher Scientific (Pittsburgh, PA, USA). Dry solvents were prepared by a Vigor Tech purification system (Houston, TX, USA). Certificated pure CO calibration gas was purchased from GASCO (Oldsmar, FL, USA). UV−vis absorption spectra were obtained by using a Shimadzu PharmaSpec UV-1700 UV−visible spectrophotometer (Kyoto, Japan). Fluorescence spectra were recorded on a Shimadzu RF5301PC fluorometer (Kyoto, Japan). ^1^H NMR (400 MHz) and ^13^C NMR (101 MHz) were acquired on a Bruker AV-400 MHz Ultra Shield NMR(Billerica, MA, USA).

### 3.2. Synthesis of the Nap-BC

Nap-BC was synthesized following the original literature procedure [21]. Detailed procedures and structural characterizations are described in the Supporting Information file.

### 3.3. Spectroscopic Experiments of Nap-BC

UV and fluorescence experiments of Nap-BC (10 μM, CH_3_CN-H_2_O (*v*/*v*, 4:1)) were carried out at room temperature. The fluorometer instrument parameters were set as λ_ex_ = 445 nm, 5.0 nm excitation slit width, 5.0 nm emission slit width, and low-sensitivity mode of detection.

Nap-BC stock solution was prepared in CH_3_CN-H_2_O (*v*/*v*, 4:1) at a concentration of 0.5 mM. The Cu(II) (1 mM) solution was prepared by dissolving the metal ion in deionized water. All Nap-BC solutions for spectroscopic experiments were prepared in a CH_3_CN-H_2_O (*v*/*v*, 4:1) with a final concentration of 10 μM. As an example, 784 μL of CH_3_CN, 196 μL of deionized water, and 20 μL of Nap-BC stock solution (0.5 mM) were added to a 1.5 mL cuvette to give a 10 μM Nap-BC solution.

All experiments were triplicated.

### 3.4. Effects of CO Gas on the Fluorescence of Nap-BC

For the effects of CO gas on the fluorescence of Nap-BC-Cu(II) (10 μM), both CO balloon (around 250 mL) and CO gas bubbling methods were used with an exposure time of 15 min or 60 min. As an example for the CO bubbling experiment, 1 mL of the Nap-BC-Cu(II) mixture (10 μM) was placed in the fluorescence cuvette. Then, pure CO gas was directly bubbled into the cuvette with a moderate flow rate through a long syringe needle (the pressure of CO gas tank is around 10 psi) for 15 min. Subsequently, fluorescence data were recorded.

The Nap-BC-Cu(II) mixture (2 mL, 10 μM) in a 20 mL vial was subjected to three cycles of evacuation and refilling with carbon monoxide (CO) gas before being attached to a CO ballon with around 250 mL pure CO. The mixture was stirred at 800 rpm for 15 min. Subsequently, fluorescence measurements were carried out [27].

## 4. Conclusions

In summary, we synthesized and re-evaluated a reported copper-bridged fluorescent system Nap-BC-Cu(II) for CO detection. In our experiments, CORM-3 led to some fluorescence restoration (20% at the 15 min point). Exposure of Nap-BC-Cu(II) to CO in the form of a CO balloon (1 million-fold excess) with stirring did not lead to any meaningful fluorescence changes. Only vigorous bubbling of CO for more than 15 min led to a small, but unmistakable level of fluorescence restoration (10%). Though this is not a condition that is within the context of studying CO biology, there could be interesting chemistry questions for further explorations. Furthermore, a binary sensing system has to consider the association constant in solution between the two components: Cu(II) and Nap-BC. Copper toxicity is another issue. Overall, our findings do not support the notion of Nap-BC-Cu(II) being a CO probe for studying CO biology because of the lack of sensitivity and selectivity.

## Data Availability

The authors declare that all data supporting the findings of this study are included within the article and its Supplementary Information files. Raw data in alternative formats are available from the corresponding author upon reasonable request.

## Supplementary Materials

The following supporting information can be downloaded at: https://www.mdpi.com/article/10.3390/molecules31030415/s1, Figure S1: ^1^H NMR spectrum of Nap-BC in DMSO-*d_6_*. Figure S2: ^13^C NMR spectrum of Nap-BC in DMSO-*d*_6_. Figure S3: (**A**). UV-Vis spectra of Nap-BC at 10, 20, 40 mM in CH_3_CN-H_2_O (*v*/*v*, 4:1) solution. (**B**). Fluorescence stability studies of Nap-BC and Nap-BC-Cu(II). Figure S4: (**A**). UV-Vis spectra of Nap-BC at 10, 20, 40 mM in CH_3_CN-H_2_O (*v*/*v*, 4:1) solution. (**B**). Fluorescence stability studies of Nap-BC and Nap-BC-Cu(II). Figure S5: (**A**). Fluorescence responses of the Nap-BC-Cu(II) system (10 μM) to CO saturated solution at different time points. (**B**). Fluorescence responses of the Nap-BC-Cu(II) system (10 μM) to 100 μM GSH at room temperature. (λ_ex_= 445 nm, slit width= 5 nm). Figure S6: Fluorescence responses of Nap-BC (10 μM) to excess amount of freshly made CuCl (around 440 μM in the system, not totally dissolved) (λ_ex_ = 445 nm, slit width= 5 nm). Reference [21] is cited in the supplementary materials.
