# Supplementary material for: A Cu(II)-Based Fluorescent Probe for Carbon Monoxide, Nap-BC-Cu(II), Does Not Selectively Detect Carbon Monoxide
Source: Molecules. 2026 Jan 26;31(3):415. doi: 10.3390/molecules31030415 (PMC12898799; doi:10.3390/molecules31030415)
Supplement: Supplementary file 1 [file molecules-31-00415-s001.zip › molecules-4087644-supplementary.pdf]

Supplemental Information

A Cu(II)-Based Fluorescent Probe for Carbon Monoxide, Nap-BC-Cu(II), Does Not Selectively Detect Carbon Monoxide

Dongning Liu, Hongliang Li, Shivanagababu Challa and Binghe Wang*

Department of Chemistry and Center for Diagnostics and Therapeutics, Georgia State University, Atlanta, Georgia 30303, United States

*Email: wang@gsu.edu.

Content

Experimental Section **S2**

Supporting Figures **S3**

References **S8**

Experimental Section

Material and Instruments

Chemical reagents were purchased from Sigma-Aldrich (Saint Louis, MO) and/or Oakwood (Estill, SC). Solvents were purchased from Fisher Scientific (Pittsburgh, PA). Dry solvents were prepared by a Vigor Tech purification system (Houston, TX). Certified pure CO calibration gas was purchased from GASCO (Oldsmar, FL). UV−vis absorption spectra were obtained by using a Shimadzu PharmaSpec UV-1700 UV−visible spectrophotometer (Kyoto, Japan). Fluorescence spectra were recorded on a Shimadzu RF5301PC fluorometer (Kyoto, Japan). ^{1}H NMR (400 MHz) and ^{13}C NMR (101 MHz) were acquired on a Bruker AV-400 MHz Ultra Shield NMR.

Synthesis of the Nap-BC

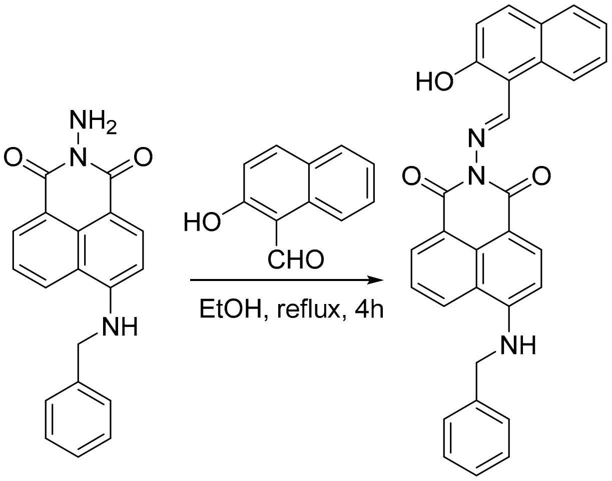

Compound 1 **Nap-BC**

The synthesis of Nap-BC was carried out following the procedure reported in the original publication.[1] Specifically, a mixture of **compound 1** (90 mg, 0.28 mmol), 2-hydroxy-1-naphthaldehyde (52 mg, 0.30 mmol), and anhydrous ethanol (15 mL) were added to a 100 mL round-bottom flask. The reaction mixture was heated to reflux for 4 h. Completion of the reaction was confirmed by TLC analysis. Afterward, the heat source was removed, and the mixture was allowed to cool to room temperature. The resulting solid was collected by filtration and washed with anhydrous ethanol (30 mL × 3). The product was dried under vacuum to obtain **Nap-BC** as an orange solid (21 mg, 14% yield). ^{1}H NMR (400 MHz, DMSO-d_6) δ 12.68 (s, 1H), 9.77 (s, 1H), 8.86 (d, J = 8.9 Hz, 1H), 8.69 – 8.62 (m, 1H), 8.56 (d, J = 7.3 Hz, 1H), 8.46 (d, J = 8.4 Hz, 1H),

8.27 (d, J = 8.8 Hz, 1H), 8.09 (d, J = 9.0 Hz, 1H), 7.94 (d, J = 8.1 Hz, 1H), 7.83 – 7.78 (m, 1H), 7.63 – 7.57 (m, 1H), 7.48 – 7.39 (m, 3H), 7.38 – 7.23 (m, 4H), 6.75 (d, J = 8.8 Hz, 1H), 4.71 (d, J = 3.9 Hz, 2H). ^{13}C NMR (101 MHz, DMSO-d_6) δ 169.4, 161.2, 160.8, 160.6, 151.4, 138.7, 135.1, 132.7, 131.8, 129.4, 129.0, 128.8, 127.6, 127.4, 125.2, 124.4, 122.6, 122.2, 120.9, 119.3, 108.6, 108.3, 105.3, 46.4. ESI-HRMS ($C_{30}H_{22}N_3O_3$, m/z): $[M+H]^+$ calculated: 472.1661, found: 472.1647.

Experimental Procedure:

Preparation of fresh CuCl solution

$CuCl_2$ (3 mg, 0.022 mmol) was dissolved in 1 mL of a mixed acetonitrile/water solvent (ACN/H_2O = 4:1, v/v). Sodium ascorbate (9 mg, 0.045 mmol, 2 equiv.) was then added to the solution. After vortexing for 10 s, the solution changed from blue to cloudy white, indicating the reduction of Cu(II) to Cu(I). 10 μL of this reaction mixture was directly added to a Nap-BC solution (10 μM) to provide an excessive amount of CuCl. Fluorescence measurements were performed immediately thereafter.

Supporting Figures

NMR spectra

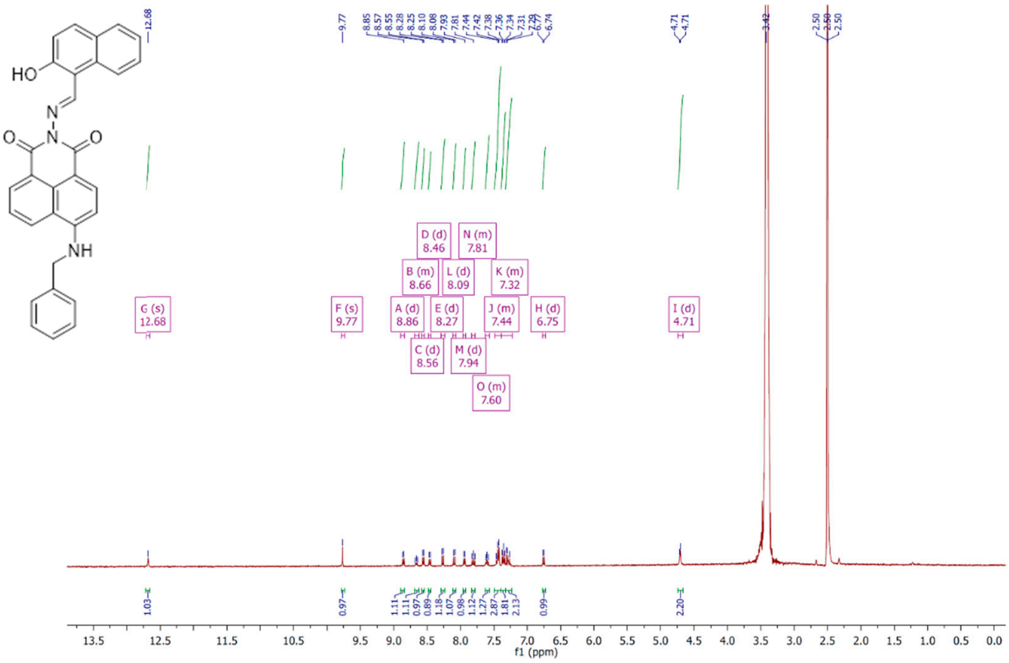

Figure S1. ^{1}H NMR spectrum of Nap-BC in DMSO-*d6*.

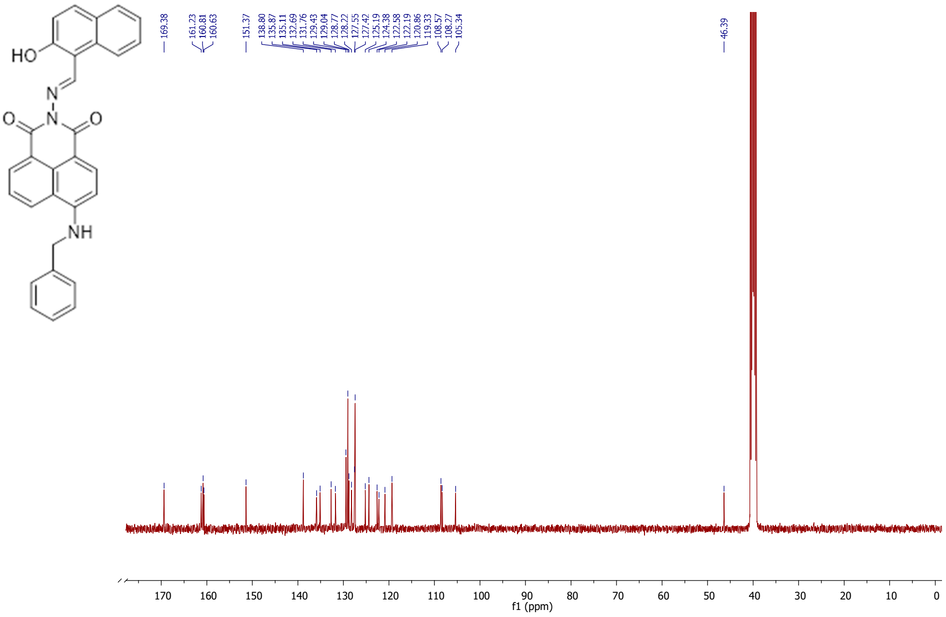

Figure S2. ^{13}C NMR spectrum of Nap-BC in DMSO-*d6*.

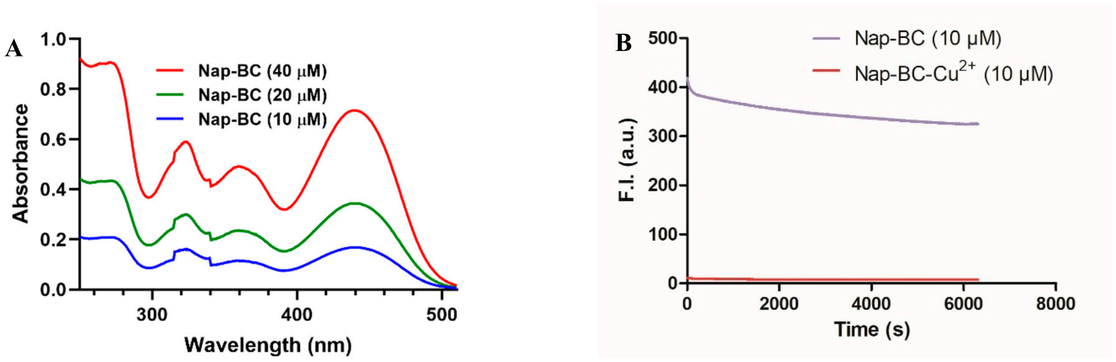

Figure S3. **A**. UV-Vis spectra of Nap-BC at 10, 20, 40 µM in CH_3CN-H_2O (v/v, 4:1) solution. **B**. Fluorescence stability studies of Nap-BC and Nap-BC-Cu(II).

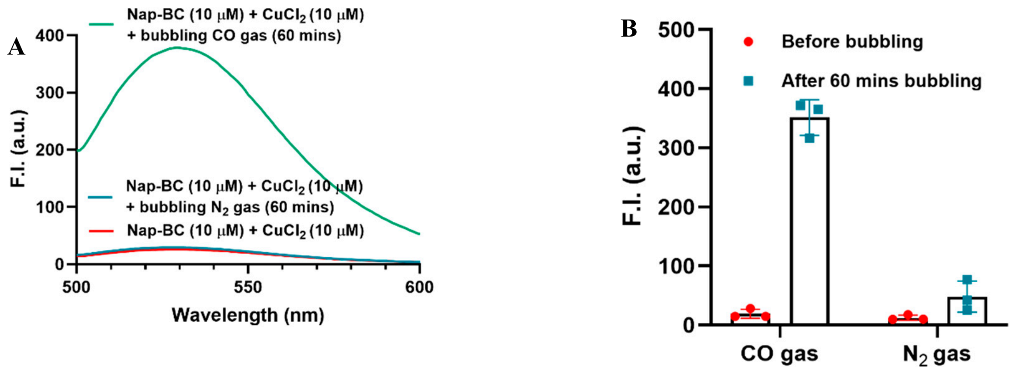

Figure S4. **A.** Fluorescence responses of the Nap-BC-Cu(II) system (10 µM) under bubbling CO or N_2 gas for 60 mins. **B.** Comparison of fluorescence intensity after bubbling CO or N_2 gas for 60 mins. (λ_{ex}= 445 nm, slit width= 5 nm).

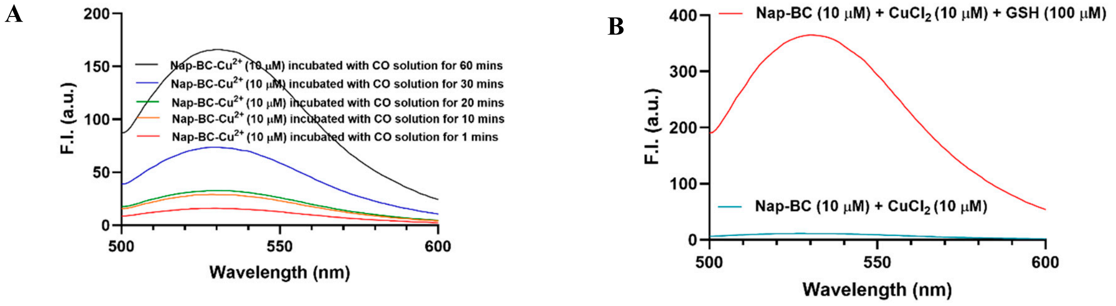

Figure S5. **A.** Fluorescence responses of the Nap-BC-Cu(II) system (10 µM) to CO saturated solution at different time points. **B.** Fluorescence responses of the Nap-BC-Cu(II) system (10 µM) to 100 µM GSH at room temperature. (λ_{ex}= 445 nm, slit width= 5 nm).

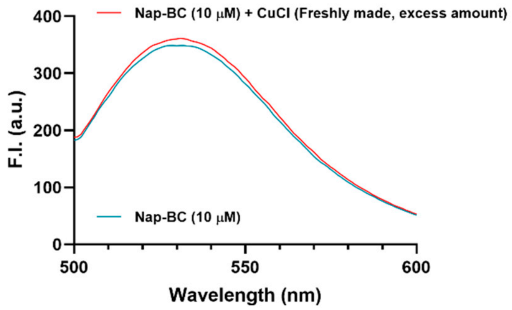

Figure S6. Fluorescence responses of Nap-BC (10 μM) to excess amount of freshly made CuCl (around 440 μM in the system, not totally dissolved) (λ_{ex}= 445 nm, slit width= 5 nm).

Reference

1. Fang, X.; Yue, B.; Wang, W.; Liu, Q.; Wu, S.; Zhang, L.; Yu, H.; Qi, Y., Copper-bridged fluorescent turn-on molecular system for high-performance identification and bioimaging of carbon monoxide. *Sensors and Actuators B: Chemical* **2024,** *421*, 136542.
